# Therapeutic potential of histone deacetylase inhibitor for treatment of Niemann-Pick type C1 disease

**DOI:** 10.1186/1755-8166-7-S1-O2

**Published:** 2014-01-21

**Authors:** Nina H Pipalia, Frederick R Maxfield

**Affiliations:** 1 Department of Biochemistry, Weill Cornell medical College, 1300 York Ave. New York, NY 10065, USA

## Background

Niemann-Pick type C (NPC) is a rare, autosomal recessive neurodegenerative lysosomal storage disorder (LSD) caused due to mutation in either *npc1* or *npc2* genes. However, 95% of the reported cases are caused due to mutation in *npc1* gene and only 5% due to the mutation in *npc2* gene. Mutations in either of these two genes results in similar phenotype such that there is an abnormal accumulation of unesterified cholesterol and glycosphingolipids in late endosomes/ lysosomes (LE/Ly) of many cell types. NPC1 is a multi-spanning transmembrane protein localized in limiting membrane of LE/Ly whereas NPC2 is soluble cholesterol binding protein localized in the lumen of LE/Ly. There are no therapeutic options to treat the disease and the children born with this defect die before the age of 20 years. Miglustat, a drug used to treat Gaucher disease has been reported to stabilize NPC patients and has also been approved for treating NPC patients in Europe. Infusion of very high dose of chemical chaperone like 2-hydroxypropyl-β-cyclodextrin (HPBCD) several times a week has also been shown to reduce the cholesterol load in peripheral tissues. Unfortunately, the blood brain barrier (BBB) cross over capability of HPCD is very poor and hence requires intrathecal CNS injections.

## Results

We have found that the treatment of several histone deacetylase inhibitors (HDACi), corrects the NPC defect specifically in human patient derived NPC1, but not in NPC2 fibroblast. Amongst the HDACi tested Vorinostat is a FDA approved drug that has been shown to cross BBB and is currently used in clinic for treatment of cutaneous T-cell lymphoma and many other type of cancer. Our initial study was conducted on most common mutation “I1061T” found in human patients but since then we have tested the effect of Vorinostat and other HDACi’s on several different patient derived mutant fibroblasts.

## Conclusions

Our data indicate that Vorinostat is effective in rescuing the phenotype in most cases but to a varying degree. This is a promising breakthrough example of repurposing existing FDA approved drug for the treatment of NPC1 and other LSDs.

